# A Comparison of B16 Melanoma Cells and 3T3 Fibroblasts Concerning Cell Viability and ROS Production in the Presence of Melatonin, Tested Over a Wide Range of Concentrations

**DOI:** 10.3390/ijms14023901

**Published:** 2013-02-14

**Authors:** Maria Angeles Bonmati-Carrion, Nuria Álvarez-Sánchez, Rüdiger Hardeland, Juan Antonio Madrid, Maria Angeles Rol

**Affiliations:** 1 Department of Physiology, College of Biology, University of Murcia, Murcia 30100, Spain; E-Mails: mbc11365@um.es (M.A.B.-C.); angerol@um.es (M.A.R.); 2 Institute of Biomedicine of Seville (IBiS), Virgen del Rocío University Hospital/CSIC/University of Seville, Seville 41013, Spain; E-Mail: nalvarez-ibis@us.es; 3 Department of Medical Biochemistry and Molecular Biology, University of Seville School of Medicine, Seville 41004, Spain; 4 Johann Friedrich Blumenbach Institute of Zoology and Anthropology, University of Göttingen, Göttingen 37077, Germany; E-Mail: rhardel@gwdg.de

**Keywords:** melanoma, fibroblast, melatonin, cell viability, intracellular ROS, tumor cell cultures, non-tumor cell cultures, *in vitro*

## Abstract

Melatonin is a pleiotropic molecule with many cellular and systemic actions, including chronobiotic effects. Beneficial effects are widely documented concerning the treatment of neoplastic diseases *in vivo* as well as reductions in viability of cultured cells from melanoma, one of the most aggressive cancers in humans. However, studies of its effects on non-tumor cells *in vitro* have not focused on viability, except for experiments aiming to protect against oxidotoxicity or other toxicological insults. Furthermore, there is no agreement on the range of effective melatonin concentrations *in vitro*, and the mechanisms that reduce cell viability have remained unclear. Tumor cell-specific increases in the production of reactive oxygen and nitrogen species (ROS/RNS) may provide a possible explanation. Our aim was to analyze the potential inhibition of tumor (B16 melanoma 4A5) and non-tumor cell (3T3 Swiss albino) viability using a wide range of melatonin concentrations (10^−11^–10^−2^ M), and to determine whether intracellular ROS enhancement was involved in this process. In the absence of fetal bovine serum (FBS), low melatonin concentrations (10^−9^–10^−5^ M) reduced the proliferation of melanoma cells with no effect in fibroblasts, whereas, in the presence of FBS, they had no effect or even increased the proliferation of both fibroblast and melanoma cells. Melatonin concentrations in the upper millimolar range increased ROS levels and reduced the viability of both cell types, but more markedly so in non-tumor cells. Thus, low melatonin concentrations reduce proliferation in this specific melanoma cell line, whereas high concentrations affect the viability of both tumor (B16 4A5 melanoma) and non-tumor (3T3 fibroblasts) cells. Increased ROS levels in both lines indicate a role for ROS production in the reduction of cell viability at high—but not low—melatonin concentrations, although the mechanism of action still remains to be elucidated.

## 1. Introduction

Melatonin (*N*-acetyl-5-methoxytryptamine) is a hormone that is synthesized and secreted during the dark phase of the day by the pineal gland. Moreover, an increasing body of evidence shows that it is also produced in various extrapineal organs, including the retina, gastrointestinal tract, bone marrow, lymphocytes [1,2], and heart [3], among others. In mammals, the nocturnal rise is controlled by the hypothalamic circadian pacemaker, the suprachiasmatic nuclei (SCN), which triggers sympathetic stimulation of the pineal gland via a specific neuronal pathway, whereas melatonin biosynthesis is inhibited by light. Photic information is transmitted to the SCN through the retinohypothalamic tract [4].

In addition to its properties of a chronobiotic agent, melatonin acts as a pleiotropic regulator that exerts a plethora of cellular and systemic effects [2,5]. Among other actions, it is now accepted that it has immune-enhancing [6], antioxidant [7] and oncostatic properties [8]. Antitumor effects of melatonin have been explored both *in vivo* and *in vitro*. In fact, melatonin has been widely documented to be beneficial in various cases of neoplastic diseases *in vivo* [9], including melanoma [8], one of the most invasive cancers in humans [10]. Although various suggestions concerning melatonergic signaling pathways and metabolic changes have been published for several tumors and respective cell lines [11–15], it remains to be clarified whether oncostatic and oncocidal effects, when present, are directly caused by the hormone *via* binding sites in the tumor cells, indirectly *via* modulation of the circadian and/or the immune systems or by several concomitant actions. A further problem with *in vivo* systems consists in the frequently observed interference of melatonin with other signals (e.g., by hormones, cytokines, cell-cell interactions or fatty acid uptake), making it difficult to interpret the results. For these reasons, among others, *in vitro* studies have been proposed as a useful tool to investigate the effects of melatonin and other agents.

The effects of melatonin on the viability of cultured tumor cells are documented in an extensive body of literature. Some examples are hepatocellular carcinomas [16,17], lymphomas [18], mammary tumors [19–21], pituitary prolactin-secreting tumors [22], gliomas [23], pheochromocytomas [24], and melanomas [12,25–30]. In the majority of cases, melatonin was capable of reducing tumor cell viability. However, there are some examples in which melatonin had no such effect [31], and rather enhanced tumor cell proliferation or survival [24], in a certain concentration range. A moderate growth stimulation was even observed in melanoma B16 cell lines [28,32], at low doses of melatonin, whereas as inhibition was reported for another B16 subline [29]. These discrepancies are not entirely surprising, inasmuch as established cell lines, even if derived from the same carcinoma type or the same biopsy material, frequently differ substantially in their properties, because of multiple mutations and epigenetic effects by which tumor cells differently shut off certain genes. With regard to the action of a hormone such as melatonin, differences in the expression of receptors (cf. data by Helton *et al.* [28], Cos *et al.* [29]*vs.* Mengeaud *et al.* (1994) [33]) and other binding sites can lead to fundamental deviations in responsiveness between tumor cell sublines. In fact, comparisons of several human melanoma cell lines revealed considerable differences in their susceptibility to melatonin, also depending on stages and phenotypes [30]. Although *in vitro* studies are useful to avoid the above-mentioned problems of *in vivo* studies, variations among cell lines related to the state of differentiation or the number of mutations have to be taken into account, especially in the case of melanomas (cf. [30]). Corresponding assumptions are applicable to tumors *in vivo* and to comparisons of *in vitro* with *in vivo* studies [8,28].

With regard to this variability, there is no agreement on the concentrations of melatonin required for efficiently reducing cell viability *in vitro*. Some reports indicate that low concentrations are effective in melanoma cultures [29] and in other tumor cell lines [19], while other studies suggest that very high concentrations are required [17]. In addition, fetal bovine serum (FBS), a necessary component of culture media for most cell lines, has been reported to interfere with the action of melatonin, lowering its effectiveness in reducing cell viability [18,30].

The mechanisms involved in the action of melatonin on cell viability remain to be identified. Suggestions have been made concerning a role for melatonin receptors [17,21,34], the activation of several apoptotic pathways and cell cycle modifications [16,22]. Although melatonin is a potent radical scavenger and antioxidant at different levels, e.g., by increasing the activities of antioxidant enzymes [35,36] or by modulating mitochondrial activity (for a review, see ref. [37]), stimulations of ROS/RNS production have been also documented in some cell types, at both pharmacological and physiological concentrations. This is particularly evident in the immune system, in which melatonin directly activates monocytes [38–40]. However, most of the prooxidant effects are of indirect nature and related to the stimulation of proinflammatory cytokine secretion (IL-2, IL-6, IL-8, IL-12) [2,6,41] or suppression of the anti-inflammatory IL-10 [42].

Although melatonin exhibits antiapoptotic properties in numerous models in which cells are toxicologically challenged [43], the contrary has been repeatedly described for cancer cells [44,45]. In the scientific literature of recent years, the hypothesis has gained increasing support that melatonin can also reduce cell viability and activate cell death pathways by enhancing intracellular ROS formation, particularly in cultured tumor cell lines [45–48]. Surprisingly, in non-tumor cell lines such as human leukocytes, it is the antioxidant effect of melatonin, at physiological concentrations, that are responsible for its protective actions [43].

Melatonin has been proposed as a potentially safe and effective oncostatic agent in cancer patients. However, and although many studies have investigated the effects of melatonin on non-tumor cells [49–52], they have not focused strictly on cell viability but rather on other aspects of melatonin actions, such as paracrine interactions [53,54]. Apart from controls in toxicological studies or protection experiments against UVA and UVB radiation [55], only a few investigations have dealt with cell viability in non-tumor cell cultures. However, to the best of our knowledge, no single study has ever compared its effects on the viability of tumor and non-tumor cell lines *in vitro* using identical melatonin concentrations, culture media composition and exposure times.

Therefore, the aim of this study was to investigate the possible differential effect of melatonin in a tumor and a non-tumor cell line, using a wide range of melatonin concentrations, to examine the interferential effect exerted by FBS on the action of melatonin, and to determine whether the enhancing effect of high melatonin concentrations on intracellular ROS constitutes a possible mechanism underlying the reduction of cell viability.

## 2. Results and Discussion

### 2.1. Results

**Figure 1:**
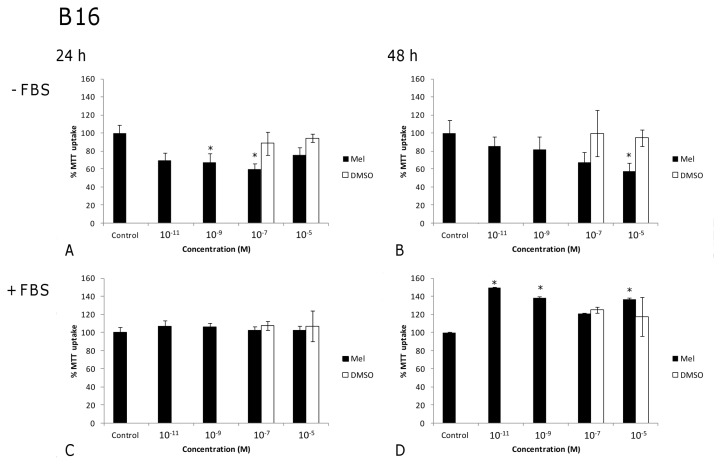
Melanoma cell line (B16): Effects of melatonin on counts of viable cells (expressed as the percentage of MTT uptake with respect to the untreated control), in a low concentration range, in the absence (**A** and **B**) or the presence (**C** and **D**) of fetal bovine serum (FBS) after 24 (**A** and **C**) and 48 h (**B** and **D**) of treatment. DMSO vehicle controls were performed for each melatonin concentration, although for terms of clarity only the two highest concentrations are represented. Statistically significant differences compared to controls are marked with asterisks. (^*^ indicates *p* < 0.05, ANOVA followed by Dunnett’s *post hoc* test).

**Figure 2:**
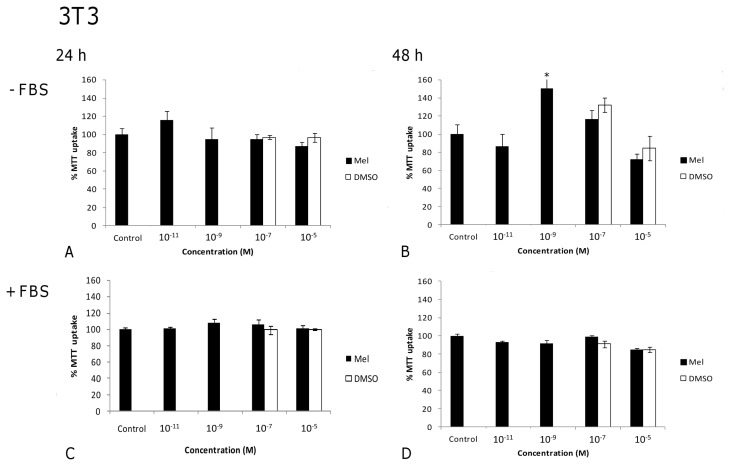
Fibroblast cell line (3T3): Effects of melatonin on counts of viable cells (expressed as the percentage of MTT uptake with respect to the untreated control), in a low concentration range, in the absence (**A** and **B**) or the presence (**C** and **D**) of fetal bovine serum (FBS) after 24 (**A** and **C**) and 48 h (**B** and **D**) of treatment. DMSO vehicle controls were performed for each melatonin concentration, although for terms of clarity only the two highest concentrations are represented. Statistically significant differences compared to controls are marked with asterisks. (^*^ indicates *p* < 0.05, ANOVA followed by Dunnett’s *post hoc* test).

**Figure 3:**
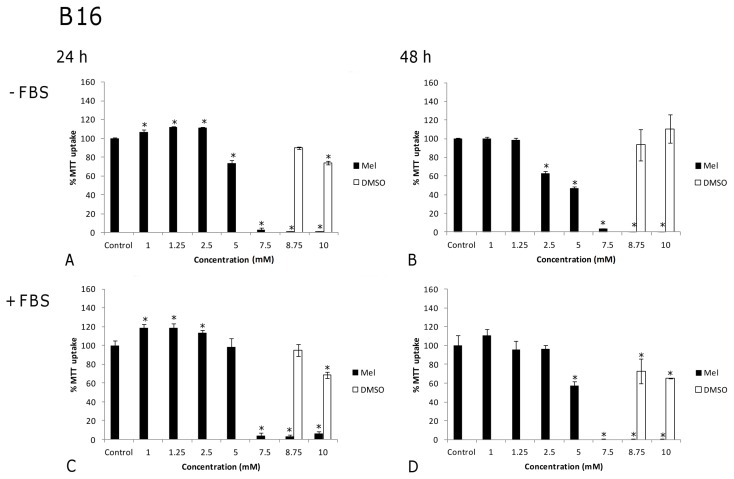
Melanoma cell line (B16): Effects of melatonin on cell viability (expressed as the percentage of MTT uptake with respect to the untreated control), in a high concentration range, in the absence (**A** and **B**) or the presence (**C** and **D**) of fetal bovine serum (FBS) after 24 (**A** and **C**) and 48 h (**B** and **D**) of treatment. DMSO vehicle controls were performed for each melatonin concentration, although for terms of clarity only the two highest concentrations are represented. Statistically significant differences compared to controls are marked with asterisks. (^*^ indicates *p* < 0.05, ANOVA followed by Dunnett’s *post hoc* test).

**Figure 4:**
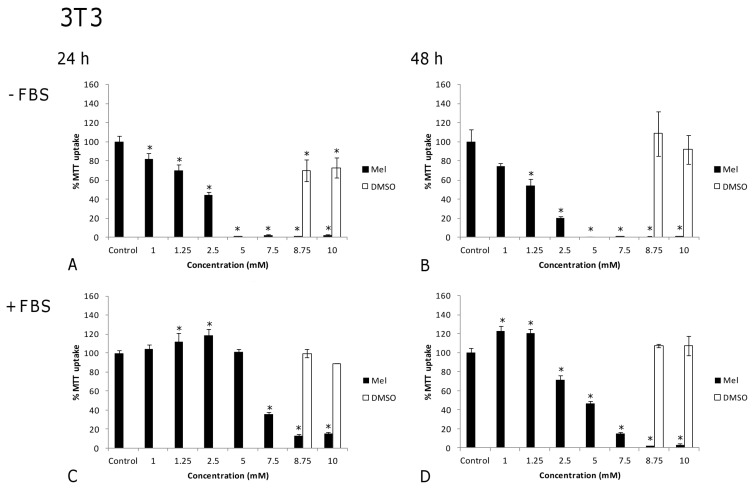
Fibroblast cell line (3T3): Effects of melatonin on cell viability (expressed as the percentage of MTT uptake with respect to the untreated control), in a high concentration range, in the absence (**A** and **B**) or the presence (**C** and **D**) of fetal bovine serum (FBS) after 24 (**A** and **C**) and 48 h (**B** and **D**) of treatment. DMSO vehicle controls were performed for each melatonin concentration, although for terms of clarity only the two highest concentrations are represented. Statistically significant differences compared to controls are marked with asterisks. (^*^ indicates *p* < 0.05, ANOVA followed by Dunnett’s *post hoc* test).

The most relevant results concerning the effects of melatonin on cell viability are shown in Figures 1, 2, 3 and 4. In the absence of FBS, low melatonin concentrations ranging from 10^−9^ to 10^−5^ M reduced moderately, but significantly, counts of viable B16 melanoma cells (Figure 1A,B), as compared to untreated cells. However, when FBS was present (Figure 1C,D), low-range melatonin concentrations (10^−11^–10^−5^) had no effect at 24 h, and significantly increased cell numbers after 48 h (with the exception of 10^−7^ M, which did not reach significance).

In the case of fibroblast (non-tumor) 3T3 cells, no obvious tendency was seen for this same concentration range in terms of reducing cell numbers in the absence of FBS at either 24 h or 48 h (Figure 2A,B). After 48 h of treatment, a non-significant 30% reduction was found at the highest concentration (10^−5^ M) (Figure 2B), whereas 10^−9^ M melatonin caused a significant increase in cell number (*p* < 0.004). On the other hand, in the presence of FBS, and with this low range of melatonin concentration, practically no changes in cell viability were found after 24 or 48 h of exposure (Figure 2C,D). Thus, in the absence of FBS, low melatonin concentrations (10^−11^–10^−5^ M) significantly reduced the number of viable cells of the melanoma line, without affecting or even increasing that of the fibroblast cell line.

**Figure 5:**
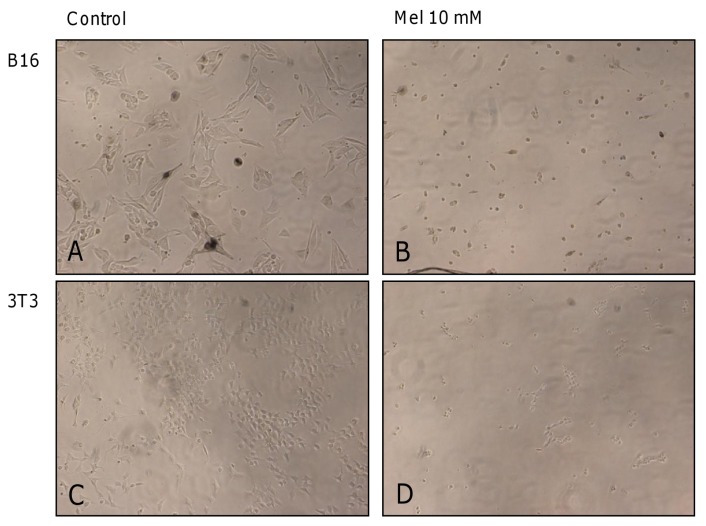
Lethal effect on melanoma (B16) (**A** and **B**) and fibroblast cell lines (3T3) (**C** and **D**) in the presence of FBS. Microphotographs in A and C represent untreated control cells. Microphotographs B and D are taken from cell cultures treated with 10 mM melatonin after 48 h. A loss of cell structure can be clearly observed. The images were obtained using a 20× objective.

Figure 3 shows the effects by a range of high melatonin concentrations (1–10 mM), with and without FBS in the culture medium, on the melanoma cell line after 24 h and 48 h of exposure. In the absence of FBS and after 24 h of treatment, a 20% reduction was observed in the number of viable cells, at a melatonin concentration of 5 mM (Figure 3A, *p* < 0.001). After 48 h, however, cell viability compared to untreated control cells was significantly lowered by melatonin concentrations above 2.5 mM, with reductions of 40% and 50% following the administration of 2.5 mM and 5 mM melatonin, respectively (Figure 3B, *p* < 0.001). At concentrations close to 8.75 mM, melatonin became strongly lethal. Cells exhibited a completely disorganized structure and their viability was reduced to 0% (Figure 3A,B, *p* < 0.001). In the presence of FBS (Figure 3C,D), a 5 mM melatonin concentration was needed to produce a 40% reduction in cell viability after 48 h of exposure (Figure 3D, *p* < 0.001), which is twice the concentration needed in the absence of FBS; at the same time, the lethal concentration decreased to 7.5 mM (Figure 3D, *p* < 0.001) (Figure 5A,B).

When melatonin was applied in the millimolar range to 3T3 fibroblasts in the absence of FBS, cell viability declined with increasing melatonin concentrations. At 24 h, differences to controls became statistically significant at 1 mM (Figure 4A, 20%, *p* = 0.007). Similar results were obtained after 48 h of treatment (Figure 4B). At a concentration of 5 mM, melatonin became lethal after 24 h or 48 h of treatment (*p* < 0.001). However, in the presence of FBS, 7.5 mM melatonin was needed in this cell line to elicit a reduction in cell viability (around 60%) after 24 h of exposure (Figure 4C, *p* < 0.001). After 48 h (Figure 4D), this inhibitory effect became statistically significant (*p* = 0.001) at lower melatonin concentrations (2.5 mM). At 8.75 mM, melatonin turned out to be completely lethal after 48 h (*p* < 0.001), losses in viability being also apparent in damaged cell structure (Figures 4D,5C,D). In summary, 3T3 fibroblasts were more sensitive to high concentrations of melatonin in the absence of FBS, but not in its presence.

In order to ascertain that the effect of melatonin was not merely due to an increase in osmolarity or to a toxic effect by DMSO, controls for DMSO vehicle (Figures 1, 2, 3 and 4) and osmolarity (data not shown) (using sucrose to match the osmolarity of melatonin solutions, as detailed in Table 1) were also included. No differences were observed when comparing cells treated with DMSO or sucrose at different concentrations to non-treated controls in either tumor or non-tumor cells at 24 h or 48 h. In fact, the increase in osmolarity in melatonin solutions was due to DMSO, in such a way that the vehicle-treated cells could easily serve as osmolarity controls (Table 1). This clearly demonstrates that the changes observed in cell viability result from melatonin treatment but not from an inappropriately high osmolarity or from DMSO toxicity.

**Figure 6:**
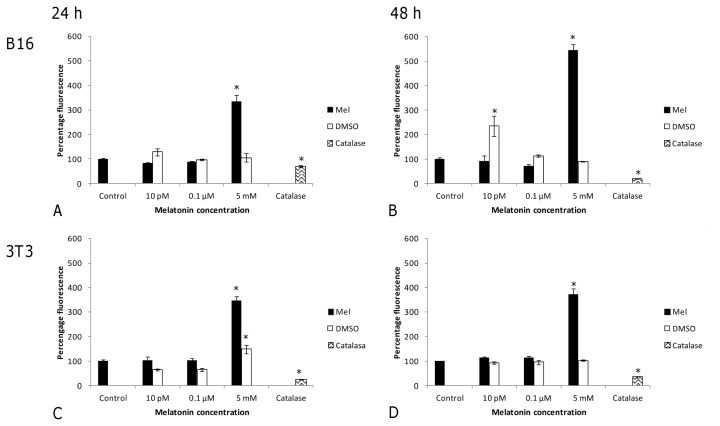
Intracellular DCFH-reactive ROS levels in melanoma (**A** and **B**) and fibroblast (**C** and **D**) cell lines treated with melatonin, in the presence of FBS, and their respective controls. DCFH-reactive ROS are expressed as the percentage of green fluorescence in the presence of melatonin at three different concentrations (10 pM, 0.1 μM, 5 mM) after 24 h (**A** and **C**) and 48 h (**B** and **D**). Catalase (1000 U/mL) was used as a positive control for antioxidant activity. Statistically significant differences compared to controls are marked with asterisks. (^*^ indicates *p* < 0.05, ANOVA followed by Dunnett’s *post hoc* test).

To examine whether an intracellular increase in ROS induced by melatonin might be causative for a reduction in cell viability, the effect of three melatonin concentrations (10 pM, 0.1 μM and 5 mM) on intracellular dichlorofluorescin **(**DCFH)-reactive ROS levels was studied in both cell lines after 24 h and 48 h of treatment (Figure 6). Since flow cytometry requires cell integrity, tests were performed with non-lethal melatonin concentrations and in the presence of FBS (medium component that satisfies specific metabolic requirements for the culture of cells). The highest concentration tested (5 mM) significantly increased intracellular DCFH-reactive ROS levels in both tumor and non-tumor cell lines. In the case of melanoma cells, intracellular DCFH-reactive ROS levels increased by 334% (Figure 6A, *p* < 0.001) and 545% (Figure 6B, *p* < 0.001) after 24 h and 48 h, respectively, as compared to untreated control cells. In the fibroblast cell line, the melatonin-induced increase in intracellular DCFH-reactive ROS levels after 24 h (Figure 6C) was similar (346%) to that seen for tumor cell lines (*p* < 0.001 *vs.* untreated control), but this level was maintained (373%) even after 48 h (Figure 6D, *p* < 0.001).

Only in melanoma cells, a lower dose (10 pM) was able to produce an antioxidant effect after 48 h of exposure (Figure 6B). Catalase was used as a positive control for antioxidant activity, and as expected, it reduced intracellular DCFH-reactive ROS levels (*p* < 0.004).

### 2.2. Discussion

Our data demonstrate that melatonin affects proliferation and viability of both melanoma cells and fibroblasts differently at low and high concentrations. In the lower concentration range, *i.e.*, at physiological and moderately supraphysiological levels, the number of viable melanoma cells was transiently reduced, whereas fibroblasts remained unaffected or slightly increased in their number. The transitory nature of the inhibition already indicates that this is mainly an antiproliferative effect rather than a change in viability. This assumption is supported by the observation that the reduction in cell number is abolished by FBS. This finding confirms similar results by other laboratories obtained in various tumor cell lines. Fetal serum contains various constituents known to stimulate proliferation of melanoma cells, such as IGF-1 [56,57] and, perhaps, leptin [58], which should override minor antiproliferative effects as exerted by melatonin. If this assumption is correct, melanomas carrying properties like the B16 4A5 cells used in this study should not respond directly to physiological or low pharmacological levels of melatonin. However, indirect actions of melatonin exerted by modulation of the immune system or *via* epigenetic changes transmitted by circadian oscillators [5] may still be effective *in vivo*.

Additional effects of FBS may also reduce the actions of melatonin. Constituents of fetal serum have been discussed to also modulate the metabolism of tumor cells [30]. Another effect is of merely technical nature and consists in the sequestration of the hormone by serum proteins. In fact, intracellular melatonin concentrations were reported to be 20 times higher in the absence of FBS [18]. The sequestration by FBS likely corresponds to that by melatonin-binding plasma proteins as documented *in vivo*. All these FBS properties are in accordance with our observation that it interferes with melatonin more efficiently at low concentrations of the hormone. At elevated levels, FBS only tended to shift the inhibition by melatonin towards even higher concentrations. Independently of the mechanism involved, FBS interference has to be generally taken into account in *in vitro* studies, since it is a widely used component in many standard culture media.

Results obtained at very high melatonin concentrations strongly contrast with those from the low-dose range. Millimolar levels of melatonin clearly decreased cell viability and became lethal, in the upper range, to both melanoma cells and fibroblasts. The lethal dose was somewhat lower in non-tumor (3T3) than in melanoma cells, a finding that may have numerous reasons, which would require further studies. Small but significant differences were found between highest DMSO concentrations treated cells and untreated control cultures in some experiments, however, they could be presumably attributable to the natural DMSO citotoxicity. Our results also demonstrate that the reduction in cell viability was not due to changes in osmolarity or DMSO toxicity.

With regard to the divergent properties of melanoma cell lines, generalizations on this type of cancer should be avoided. In the B16 subline BL6, melatonin was reported to cause growth inhibition in the range of 10^−11^ to 10^−9^ M [29], whereas no such effect was observed in other B16 cells [28]. In a further study on B16 cells, with evaluations after three days, mild growth stimulation by melatonin was reported at 10^−6^ M, whereas higher concentrations were oncostatic and became, at 10^−3^ M, oncocidal [32]. This complexity becomes even more apparent, when the expression of melatonin receptors is considered. Antiproliferative effects at 10^−11^ to 10^−9^ M, as reported by Cos *et al.* (2001) [29], are in accordance with the expression of G protein-coupled melatonin receptors. In the 4A5 subline used in the present study, inhibitions at 10^−9^ to 10^−7^ M may be interpreted in a similar way, although a participation of splice variants of the putative melatonin-binding transcription factor RORα cannot be ruled out at these somewhat higher concentrations. Systematic investigations on RORα have not been conducted in B16 sublines, but its presence was shown for some, but not all, human melanoma cell lines [30]. High-affinity binding sites for melatonin were reported to be absent in the B16 subline F10 [33]. However, their expression, as demonstrated in B16 in another investigation [28], does not immediately imply growth inhibition, which was not observed in the same study. This may be interpreted in terms of deviating or defective signaling pathways. In other cases, such as the amelanotic murine melanoma cell line S91, in which growth inhibition was observed at 10^−7^ M, this may represent an action mediated by RORα, since stable transfection with the MT_1_ receptor gene (=MTNR1A) caused pronounced antiproliferative effects with an EC_50_ of 10^−10^ M [59]. Another study on S91 conducted more than one decade earlier described growth inhibition by 10^−10^ to 10^−9^ M melatonin [60]. Thus, MT_1_ expression may have been lost in this cell line prior to the later investigation.

Other discrepancies between studies may have been caused by differences in experimental conditions. In the literature, experiments vary with regard to the duration of exposure. While some investigators have used long periods of exposure to melatonin (72–240 h) [20], others have applied shorter times (12–48 h) [19]. Under varying conditions, divergent results may be obtained, as shown in the human MCF-7 breast cancer cells line. Cos *et al.* (2002) [19] observed a reduction in MCF-7 cell viability with 1 nM melatonin at 24 and 48 h, while Cucina *et al.* (2009) [20] reported that 72 h were needed for decreasing cell viability, at same concentration. In our study, similar results were obtained, for the most part, with 24 and 48 h of exposure. Especially at high melatonin concentrations, the more pronounced effects were observed with the latter period.

The sensitivity of melanoma cells to low, near-physiological concentrations of melatonin may be regarded as a primary property of this type of cancer, because the expression of high-affinity binding sites, in particular, MT_1_, was repeatedly demonstrated not only in murine, but also various hamster and human melanoma cell lines, often in conjunction with antiproliferative effects in the physiological range [25–27,61–64]. However, this sensitivity may have been lost in various melanoma cells or disappears during tumor progression, which could be a plausible explanation of the diversity of results on growth inhibition at low melatonin concentrations. Moreover, signaling pathways associated with G protein-coupled melatonin receptors are not necessarily identical with the first-discovered pertussis toxin-sensitive Gα_i_-mediated inhibition of adenylyl cyclase. In a hamster melanoma cell line, high-affinity binding of melatonin was associated with phospholipase C activation [62], which represents another, rather frequently observed signaling pathway that modulates cytoplasmic Ca^2+^ [1]. In another hamster melanoma cell line, melatonin receptors were reported to act via a cholera toxin-sensitive pathway, in conjunction with decreases in cGMP [65,66] and cADP-ribose concentrations [65] as well as enhanced ADP-ribosylation of various proteins, including the Gα_s_ subunit [67].

The majority of studies on *in vitro* actions of melatonin have been carried out in tumor cell lines. In only a few of them, the effects of melatonin on cell viability have been analyzed in non-tumor cell lines [49,50,55]. To the best of our knowledge, this is the first time that the effects on cell viability have been compared in both tumor (melanoma) and non-tumor (fibroblasts) cell lines based on same exposure times and a wide range of melatonin concentrations, in the same study. In fact, a substantial body of literature shows that melatonin is a safe oncostatic agent *in vivo*, acting specifically on tumor cells [68]. In accordance with previous studies [22,29,30,68], we have found that low melatonin concentrations are effective in moderately reducing proliferation in the tumor cell line, whereas the non-tumor cell line is not affected or even stimulated, again in accordance with other results [55].

By contrast, high melatonin concentrations dramatically decrease cell viability in both B16 melanoma cells and 3T3 fibroblasts. However, it has to be taken into account that these higher concentrations are unlikely to occur *in vivo*, and are, thus, limited to *in vitro* studies. Nonetheless, our results do highlight the importance of not extrapolating results obtained *in vitro* with high melatonin concentrations to possible *in vivo* treatments*,* as is often done when benefits are advocated in favor of the use of melatonin in tumors. On the other hand, 3T3 fibroblasts used in our study display a high proliferation rate, as is the case in most established cell lines, and, thus, share this characteristic with tumor cells. Therefore, it cannot be ruled out that the reduction in cell viability caused by high concentrations of melatonin may only occur under conditions of a fast-running cell cycle.

In mechanistic terms, the *in vitro* reduction in viability by melatonin remains to be clarified in its details. Various suggestions have been made in other studies, including the participation of melatonin receptors [21], even at high melatonin concentrations [17], the activation of different apoptotic pathways, cell cycle alterations [16,22], and cytoskeletal disorganization [69]. However, growing evidence indicates an enhanced intracellular ROS production by very high levels of melatonin, specifically in tumor cells [45,48,68]. This possibility, though not being exclusive, seems to be decisive, at least *in vitro.* Nevertheless, one should be aware that rises in ROS, as observed in our experiments, can be secondary to other processes initiating apoptotic or necrotic cell death, or represent concomitant phenomena in dying cells, especially as soon as mitochondrial electron flux is affected. At very high melatonin concentrations in the 10 mM range, the losses in cell viability are associated with the destruction of intracellular integrity. In the microphotographs presented, the lethal effect was accompanied by cytoskeletal disorganization. Indeed, melatonin has been previously described as a modulator of cytoskeletal polymerization, but several of these changes are already observed at concentrations below lethal toxicity [69]. Whether the disorganization of the cytoskeleton is primary to other toxic effects or rather, their consequence remains to be studied. Nevertheless, osmotic shock can certainly be ruled out, because similar concentrations of sucrose failed to affect cell viability or structure.

In the present study, we used an MTT technique to evaluate cell viability, and although some authors use it indistinctly to evaluate growth inhibition or cell viability [16,17,22], this assay only provides information on the activity of the mitochondrial electron transport chain in the cultured cells, but not about the mechanism responsible for the viability state (e.g., growth inhibition, apoptosis, necrosis, *etc.*), and thus apoptosis or necrosis cannot be discarded. Regardless of these possibilities, our data clearly show rises in intracellular DCFH-reactive ROS levels in response to high melatonin concentrations (5 mM) in both cell lines. This increase did not occur at lower concentrations, in accordance with the usually observed improvement of mitochondrial electron flux by moderate levels of melatonin [70,71]. Furthermore, a slight antioxidant effect was observed in B16 after 48 h of treatment.

Although the majority of authors agree that high melatonin concentrations (in the μM–mM range) are necessary to induce ROS generation [45–48], effective melatonin concentrations have not been clearly established. Furthermore, a dual effect has been reported, *i.e.*, decreased intracellular ROS levels at low melatonin concentrations, and increased levels at high concentrations [46]. This potentiating effect has also been described to be transitory, with ROS production beginning a few hours or even minutes after the addition of melatonin, and reaching a maximum level a few hours later [47]. Since the technique used in this study to determine intracellular ROS levels provides a cumulative measure of DCFH-reactive ROS, the contribution of different oxidant species remains open. However, with regard the experience that the majority of primary free oxygen radicals are superoxide anions and that these short-lived intermediates are readily transformed by SOD subforms into molecular oxygen and hydrogen peroxide, this latter species presumably represents the major fraction of DCFH-reactive ROS. Toxicity of both superoxide and hydrogen peroxide mainly depends on the formation of their more reactive derivatives, such as peroxynitrite, hydroxyl, carbonate and NO_2_ radicals [72]. Therefore, DCFH-reactive ROS represent an indicator of enhanced oxidative stress, but do not provide information on damage.

The molecular mechanisms responsible for this increased intracellular ROS levels also remain to be identified. It has been previously reported that selectively blocking each component of the melatonin receptor’s signal transduction pathways fails to prevent the ROS-potentiating effects, demonstrating that melatonin receptors do not participate in this process [39]. Another argument against receptor involvement can be deduced from the concentrations required to increase ROS levels, since they are by several orders of magnitude higher than those needed to activate cellular receptors. However, high melatonin concentrations reportedly increase MT_1_ expression, at both mRNA and protein levels, in HepG2 hepatoma cells [17].

In conclusion, we demonstrate that melatonin has different effects in fibroblasts and melanoma cells *in vitro*, with low melatonin concentrations reducing the number of viable cells in B16 cells in the absence of FBS, but causing no changes or even a slight increase in 3T3 cells. At high melatonin concentrations, the non-tumor cells were more sensitive to melatonin than the tumor cells, in terms of the lethal dose. In both cell lines, intracellular ROS levels are involved in lethal toxicity by high melatonin, although the mechanisms and the sequence of intracellular effects remain to be identified. At low concentrations, observed changes are apparently unrelated to intracellular ROS levels.

Although more studies comparing tumor and non-tumor cell lines are needed in the future, our data shed light on the necessity of avoiding extrapolations from *in vitro* results with high melatonin to potential oncostatic treatments *in vivo*.

## 3. Experimental Section

### 3.1. Cell Culture

In this study, two cell lines were used: a tumor line, the murine melanoma cell line B16Mel4A5, purchased from American Type Cell Collection (ATCC) and a non-tumor cell line, murine fibroblasts 3T3 Swiss albino, from the European Collection of Cell Cultures (ECACC). Stock cells were routinely grown as monolayer cultures in Dulbecco’s Modified Eagle’s Medium, supplemented with 100% fetal bovine serum, penicillin (100 U/mL), streptomycin (100 μg/mL) and glutamine (2 mM) in a humidified 7.5% CO_2_ atmosphere at 37 °C, changing the medium every other day. Cells were maintained in T25 (melanoma cell line) and T75 (fibroblasts cell line) flasks with a total volume of 5 and 10 mL of complete medium, respectively. They were subcultured when 90% confluence was reached. Cell viability was assessed, at this stage prior to the final viability assays, using the Trypan Blue (Sigma Chemical Co., St. Louis, MO, USA) dye exclusion method.

To exclude any possible infection by *Mycoplasma* spp., a Mycoplasma test was performed using the *Hoechst* 33258 DNA staining method. Results were negative for *Mycoplasma* spp in all the cases, so contamination by these microorganisms could be ruled out [73].

Cell culture reagents were purchased from Sigma (St. Louis, MO, USA). Culture flasks were obtained from Sarstedt (Barcelona, Spain), and dishes from Nunc® (Thermo Fisher Scientific, Madrid, Spain). Melatonin and other reagents were purchased from Sigma (Sigma, M-5250; St. Louis, MO, USA).

### 3.2. Cell Viability Assay

Both cell lines grown in complete media were replated on the appropriate 96-well plates (from passages 8–15). After 24 h (during the exponential growth phase), the medium was replaced with fresh medium containing different concentrations of melatonin: 10^−11^ M, 10^−9^ M, 10^−7^ M, 10^−5^ M (lower range), and 1 mM, 1.25 mM, 2.5 mM, 5 mM, 7.5 mM, 8.75 mM and 10 mM (higher range), dissolved in a vehicle of 0.2% DMSO. All plates were incubated in the dark, wrapped in aluminum foil to prevent the degradation of melatonin by light. In order to assure that the covering did not affect the diffusion of gases onto the plate, the pH level was measured on both covered and uncovered plates. No pH differences were found between them.

Untreated control cells were incubated with fresh culture medium, and vehicle-treated cells were exposed to the same percentage of DMSO as melatonin-treated cells. We also included osmolarity controls, using sucrose in order to reach the same osmolarity levels as in the melatonin solutions (see Table 1). The experiment was performed in 6 replicates. Each experiment was carried out in both the presence and absence of FBS to test for potential interference with the effects of melatonin. All assays were performed once the cell culture growth was exponential.

To assess the effect of melatonin on cell viability, the 3-(4,5-dimethyl-thiazol-2-yl)-2,5-diphenyl-tetrazolium bromide (MTT) cell viability assay was used as described by Denizot and Lang (1986) [74]. After treatment for 24 or 48 h with melatonin, DMSO or sucrose, the culture medium was replaced by fresh serum-free medium. MTT previously dissolved in serum-free culture medium was added to each well and incubated for 4 h. After this interval, serum-free culture media containing MTT were discarded and DMSO was added to each well to dissolve the precipitate. The optical densities were measured at a spectral wavelength of 570 nm using a microtiter plate reader (Fluostar Galaxy). To rule out any possible absorbance by melatonin, a blank with melatonin (10^−11^ M, 10^−9^ M, 10^−7^ M, 10^−5^ M, 1 mM, 1.25 mM, 2.5 mM, 5 mM, 7.5 mM, 8.75 mM and 10 mM), but without MTT and cells was also included. No differences in absorbance levels were detected when compared to a blank without melatonin.

### 3.3. ROS Production Assay

In order to evaluate the possible effect of melatonin on generation of ROS inside the cells, the 2′,7′-dichlorofluorescin diacetate (DCFH-DA) assay was used to determine the generation of ROS inside the cells. DCFH-DA permeates the cell membranes and is hydrolyzed by intracellular esterases to form dichlorofluorescin (DCFH). Subsequently, DCFH reacts with oxidizing ROS generated by intracellular stress to produce the highly fluorescent 2′,7′-dichlorofluorescein (DCF), which emits fluorescence when excited at 485 nm. Cells were treated with either different concentrations of melatonin (10 pM, 0.1 μM and 5 mM), vehicle or fresh medium, or medium with catalase (1000 U/mL) for 24 and 48 h. DCFH-DA was then added at a final concentration of 25 μM, as previously described [75]. Briefly, after being incubated in a given medium for 30 min, the cells were detached with trypsin 0.25%/EDTA 0.25% and centrifuged in a 96-well “V” bottom assay plate at 3000*g* for 10 min. The supernatant was discarded and the pellet resuspended in phosphate buffered saline (PBS). DCFH-reactive ROS produced from intracellular stress were detected using a cytometer with an argon ion laser emitting 300 mW at 488 nm, with emission measured using 525-nm band-pass filters (Coulter mod. Epics XL).

In order to rule out any possible fluorescence emission by melatonin or its metabolites [76], a blank with cells and melatonin without DCFH-DA was also included. No changes in fluorescence levels were observed.

### 3.4. Microphotographs

Cell culture photographs were taken using a camera attached to a phase contrast microscope (NIKON mod. Eclipse TE 2000).

### 3.5. Statistical Analysis

All results are expressed as mean ± SEM. Data were analyzed with SPSS^®^ v15.0 software, using a one-way ANOVA followed by Dunnett’s *post hoc* test. Values of *p* < 0.05 were considered to be statistically significant.

## 4. Conclusions

Our results confirm the effectiveness of melatonin in reducing cell viability, specifically in B16Mel4A5 melanoma cells. High concentrations of this hormone also affect cell viability of non-tumor 3T3 Swiss albino cells, already at somewhat lower doses than in the tumor cells studied. Although additional comparative studies in tumor and non-tumor cell lines are needed, we show that caution is due when extrapolating results obtained *in vitro* with high melatonin concentrations to potential oncostatic treatments *in vivo*.

## Figures and Tables

**Table 1:** Osmolality of solutions containing high melatonin concentrations.

Treatment	Osmolality (mOsm/Kg)
Control	333
10 mM	425
8.75 mM	382
7.5 mM	364
5 mM	365
2.5 mM	346
1.25 mM	341
1 mM	342
